# Development of Cancer Among Patients With Pediatric-Onset Inflammatory Bowel Disease: A Meta-analysis of Population-Based Studies

**DOI:** 10.1001/jamanetworkopen.2022.0595

**Published:** 2022-03-01

**Authors:** Rahma Elmahdi, Camilla E. Lemser, Sandra B. Thomsen, Kristine H. Allin, Manasi Agrawal, Tine Jess

**Affiliations:** 1 Center for Molecular Prediction of Inflammatory Bowel Disease, Department of Clinical Medicine, Aalborg University, Copenhagen, Denmark; 2 The Dr Henry D. Janowitz Division of Gastroenterology, Icahn School of Medicine at Mount Sinai, New York; 3 Department of Gastroenterology and Hepatology, Aalborg University Hospital, Copenhagen, Denmark

## Abstract

**Importance:**

Because the incidence of pediatric-onset inflammatory bowel disease (IBD) is increasing, knowledge of the long-term risk of cancer in this patient population is required.

**Objective:**

To evaluate the relative rate of cancer among patients with pediatric-onset IBD.

**Data Sources:**

A comprehensive systematic search was performed of MEDLINE and Embase from the date of database inception to October 31, 2021.

**Study Selection:**

All unselected, population-based cohort studies of pediatric-onset IBD assessing the risk of cancer were included. Tertiary center referrals and insurance database studies were excluded. All articles were assessed by 2 independent reviewers.

**Data Extraction and Synthesis:**

The study followed the Preferred Reporting Items for Systematic Reviews and Meta-analyses reporting guideline for data extraction and used the Newcastle-Ottawa Scale for assessment of the risk of bias and the quality of included articles.

**Main Outcomes and Measures:**

A random-effects model meta-analysis was conducted of included studies using the inverse-variance method to assess the relative rate of cancer overall and by IBD subtype (Crohn disease or ulcerative colitis), sex, and thiopurine exposure among patients with pediatric-onset IBD. Pooled relative rates (pRRs) along with 95% CIs were calculated for combined studies.

**Results:**

Of 4628 articles screened, 5 population-based studies from North America and Europe were eligible for inclusion. These studies comprised 19 812 individuals with pediatric-onset IBD followed up for 283 540 person-years in which 715 cases of cancer were identified. Meta-analysis of pRR estimates showed a 2.4-fold increased rate of cancer among patients with pediatric-onset IBD (pRR, 2.46; 95% CI, 2.06-2.93), seen among patients with Crohn disease (pRR, 2.03; 95% CI, 1.67-2.46) and those with ulcerative colitis (pRR, 2.61; 95% CI, 2.00-3.40). This increased rate is primarily due to an increased rate of liver (pRR, 55.45; 95% CI, 19.59-156.99), colorectal (pRR, 20.29; 95% CI, 15.90-25.90), and small bowel (pRR, 16.20; 95% CI, 3.52-74.66) cancers. The incidence rate of cancer among patients with pediatric-onset IBD was reported by 4 studies and ranged from 1.0 to 3.3 cases per 1000 person-years.

**Conclusions and Relevance:**

This meta-analysis of unselected, population-based studies showed a greater than 2-fold increased rate of cancer among patients with pediatric-onset IBD compared with the general pediatric populations, primarily owing to an increased rate of gastrointestinal cancers.

## Key Points

**Question:**

What is the risk of cancer among patients with pediatric-onset inflammatory bowel disease (IBD)?

**Findings:**

This meta-analysis of 5 unselected, population-based cohort studies comprising 19 812 individuals found a 2.4-fold increased rate of cancer among patients with pediatric-onset IBD, which was primarily due to gastrointestinal cancers. The absolute rate of cancer among patients with pediatric-onset IBD is low.

**Meaning:**

These results suggest that there is a greater than 2-fold increased rate of cancer among patients with pediatric-onset IBD compared with general pediatric populations, due primarily to an increased rate of gastrointestinal cancers.

## Teaser

This meta-analysis evaluates the relative rate of cancer among patients with pediatric-onset inflammatory bowel disease (IBD).

## Introduction

The hallmarks of inflammatory bowel disease (IBD), a chronic immune-mediated inflammatory disease of the gastrointestinal tract, are progressive intestinal injury and systemic inflammation, which can lead to complications such as cancer. Inflammatory bowel disease is associated with an increased risk of several types of cancer, including colon, small bowel, and upper gastrointestinal tract cancers.^1^ In contrast to the risk of cancer among adult patients with IBD, the risk of cancer among patients with pediatric-onset IBD is not well investigated, to our knowledge. Pediatric patients with IBD may have longer durations of exposure to the chronic inflammatory state and thereby a higher risk of cancer.^2^ Furthermore, among children, the incidence of IBD and the risk of cancer have increased in recent years.^3,4^

Although previous studies indicate that the risk of all-cause cancer is higher among those with pediatric-onset IBD compared with individuals without IBD, studies are heterogeneous and limited in size, and the estimates are variable.^5^ A meta-analysis of the risk of cancer among patients with pediatric-onset IBD included all studies on the subject independent of the selective nature of most studies.^6^ However, databases on smaller cohorts from referral centers are not representative for the average patient with IBD, and we are therefore unable to generalize from them to all patients with IBD. To avoid selection biases and to ensure generalizability, unselected population-based cohort studies representing all patients with IBD in a defined geographic area and over a specified period are needed. An unbiased understanding of the risk of cancer among patients with pediatric-onset IBD has implications toward long-term screening and prevention of cancer for this group.

We therefore conducted a systematic literature search and meta-analysis of population-based observational studies assessing the overall risk of cancer, the risk of cancer by site, and the risk of cancer according to IBD subtype (Crohn disease [CD] or ulcerative colitis [UC]), sex, and thiopurine use among individuals with pediatric-onset IBD compared with general pediatric populations.

## Methods

### Search Strategy

Our meta-analysis was conducted in accordance with the Preferred Reporting Items for Systematic Reviews and Meta-analyses (PRISMA) reporting guideline.^7^ We designed and executed a comprehensive systematic search using both subject headings and key words in the biomedical databases MEDLINE PubMed and Embase Ovid from the date of database inception to October 31, 2021. We included studies that reported relative risk estimates of any cancer among individuals with pediatric-onset IBD in a population-based cohort. We included peer-reviewed original research articles as well as meeting abstracts. We also searched references for all included studies as well as relevant reviews and did not implement language restrictions. Complete search terms are available in eTables 1 and 2 in the Supplement. Export and deduplication of search results were undertaken in Mendeley Reference Manager (Mendeley Ltd) and Covidence software (Covidence). This review was not registered with any register of systematic reviews.

### Eligibility Criteria

We included all studies of geographically defined population-based cohorts of individuals with pediatric-onset IBD that reported well-defined IBD diagnostic criteria and follow-up periods. *Geographically defined* indicates a state, country, or region in which all IBD diagnoses and cancer diagnoses can be captured. We excluded studies that reported data on cancer mortality alone, studies from tertiary or referral centers, and studies that combined individuals with both pediatric- and adult-onset IBD without presenting disaggregated data. In case of multiple studies reporting data from the same cohort, the order of priority for selection was the study with the longest follow-up time.

### Study Selection and Data Extraction

Study selection and data extraction were performed by 2 investigators (C.E.L. and S.B.T.) independently, in accordance with the predefined inclusion criteria. Any conflict during abstract or full-text screening was resolved through joint review or by a third arbiter (K.H.A. or T.J.). Data were extracted into Microsoft Excel (Microsoft Corp) based on guidance provided by the Cochrane Consumers and Communication Review Group’s template.^8^ Variables extracted included author(s), study group, year of publication, study start and end date, geographic location, follow-up time (in years and person-years), number of patients with IBD and reference individuals included, number of cancer events, relative risk estimates for cancer associated with pediatric-onset IBD compared with reference populations, observed incidence rates of cancer among patients with pediatric-onset IBD, sex, and medication exposure.

### Risk of Bias and Study Quality

Risk of bias and study quality were assessed using the cohort quality assessment instrument provided by the Newcastle-Ottawa Scale.^9^ Studies were assessed in 3 domains—selection, comparability, and outcome—and could be awarded a maximum total score of 9. A total score of 7 or higher suggests a high-quality study.

### Statistical Analysis

Meta-analysis was undertaken on reported adjusted relative risk estimates from each study. We calculated a pooled relative rate (pRR) summary statistic for studies included in the meta-analysis. We extracted reported adjusted overall hazard ratios or analogous estimates and accompanying 95% CIs to calculate SEs and undertake random-effects model (REM) meta-analyses using the generic inverse variance method with Hartung-Knapp-Sidik-Jonkman estimator to calculate τ^2^, a measure of study variance. We chose an REM owing to a priori assumption of the presence of both intrastudy and interstudy heterogeneity. Subgroup meta-analyses were undertaken for cancer rate by IBD subtype (CD or UC), sex, and thiopurine exposure using the same method. We additionally undertook meta-analysis for relative rate of cancer by site, if the data were available. Sensitivity meta-analyses were undertaken to include any data extracted from non–peer-reviewed publications and excluding the largest and smallest study assessed by the REM weight contribution (in percentage) to the pooled summary statistic. Publication bias was evaluated by visual inspection of a funnel plot for the degree of asymmetry. Analyses were performed in R, version 4.0.5 (R Group for Statistical Computing) using the “metagen” and “metabin” functions in “meta”^10^ and “metafor”^11^ packages.

## Results

**Figure 1.**
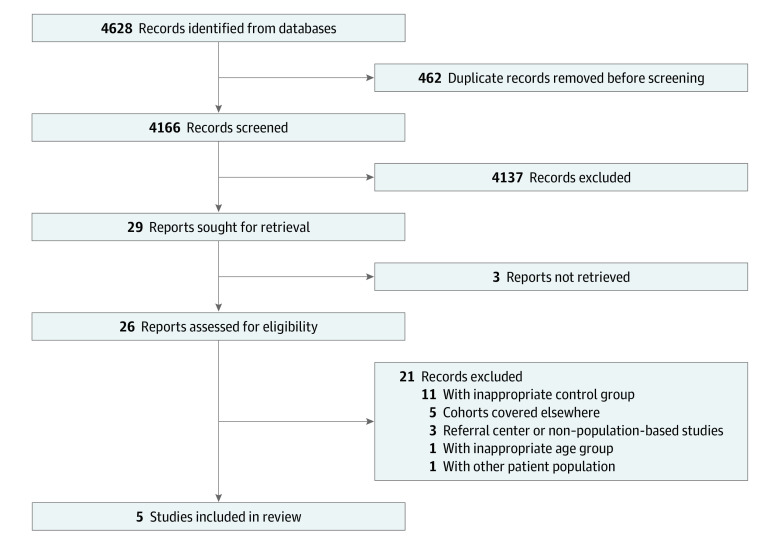
Preferred Reporting Items for Systematic Reviews and Meta-analyses Flow Chart Illustrating the Screening and Selection Process

We identified a total of 4628 abstracts from databases searched, of which 4166 remained after excluding duplicate results. Of these, a further 4140 were excluded based on title and abstract screening; 3 articles could not be retrieved, and 4137 did not meet the inclusion criteria. Twenty-six articles were reviewed in full. Twenty-one of these were excluded: 11 owing to lack of appropriate control group, 5 owing to duplicated cohorts, 3 owing to referral or tertiary center–based populations, and 2 owing to inappropriate patient population (ie, adult or other type of patient group) (Figure 1). Five full-text publications were included (Table 1)^12,13,14,15,16,17,18^: a study by El-Matary et al^12^ from Manitoba, Canada, based on the University of Manitoba IBD Epidemiology Database; a study by Peneau et al^17^ reporting on data from the population-based EPIMAD registry in northern France; a study by Olén et al^16^ that included national Swedish registry data; a study by Kjaergaard et al^14^ that included national Danish registry data; and a study by Malham et al^15^ that included both Finnish and Danish national registry data. We extracted only the Finnish data from the study by Malham et al^15^ to exclude replicating data from the study by Kjaergaard et al,^14^ which followed the same cohort for a longer duration. In addition, we included a study by Deneau and Guthery,^19^ published as a conference abstract, in a sensitivity analysis, but not in the primary analysis, owing to a lack of granular data.

**Table 1.** Characteristics of Included Articles

Source	Country	Article type	Study period	Source of cohort data	IBD population size, No.		Reference population size, No.	Cancer events in IBD population, No.		Cancer events in reference population, No.	RR estimate for cancer in pediatric-onset IBD (95% CI)	Follow-up time of IBD population, PY per year	Adjusted confounders	% of Female patients	Cancer incidence in IBD population	Median (IQR) age, y		NOS Score^a^
					CD	UC		CD	UC							At IBD diagnosis	At cancer diagnosis	
El-Matary et al,^12^ 2020	Canada	BC	1984 to 2018	University of Manitoba IBD Epidemiology Database^13^ (South-central Canada: Manitoba Province)	576	371	9272	NR	NR	75	HR, 2.00 (1.16-3.44)	14 938	Sex, age, region, or residence	NR	Incidence rate, 1.1 (per 1000 PY)	14 (12-16)	37 (24-45)	4 (Fair)
Kjaergaard et al,^14^ 2020	Denmark	BC	1977 to 2018	Danish national patient registry	2673	2707	53 800	77	81	701	HR, 2.16 (1.81-2.57)	77 821	Sex, age, calendar, and year of diagnosis	NR	Incidence rate, 2.03 (per 1000 PY)	NR	NR	8 (Good)
Malham et al,^15^ 2019	Finland	ORA	1992 to 2014	Finnish national patient registries	1261	2084	2 899 565	10	24	8160	SIR, 3.60 (2.55-5.09)	33 845; median, 9.0 (IQR, 4.4-15.0)	NR	44.4	Incidence rate, 1.0 (per 1000 PY)	14 (12-16)	23.5 (14-34)	5 (Fair)
					U-IBD = 11^b^													
Olén et al,^16^ 2017	Sweden	ORA	1964 to 2014	Swedish Patient Register	3768	4648	92 870	153	299	2256	HR, 2.20 (1.97-2.46)	148 682	Sex, age, year of birth, and region of residence	44.7	Incidence rate, 3.3 (per 1000 PY)	15 (12-16)	Not reported	9 (Good)
					U-IBD = 989^c^			U-IBD = 45^c^										
Peneau et al,^17^ 2013	France	ORA	1988 to 2004	EPIMAD registry^18^ (Northern France: Seine-Maritime, Somme, Pas-de-Calais and Nord Departements)	538	160	775	6	3	3	SIR, 3.00 (1.40-6.40)	8254; median, 11.4 (IQR, 7.4-15.8)	Sex and age	48.4	Crude cancer rate, 1.3%	14.6 (11.5-16.1)^d^	29.6 (21.5-33.1)	5 (Fair)
					U-IBD = 26^c^													

Abbreviations: BC, brief communication; CD, Crohn disease; EPIMAD, Epidémiologie des maladies inflammatoires de l’Intestin; HR, hazard ratio; IBD, inflammatory bowel disease; NOS, Newcastle-Ottawa Scale; NR, not reported; ORA, original research article; PY, person-years; RR, relative rate; SIR, standardized incidence ratio; UC, ulcerative colitis; U-IBD, unclassified IBD.

^a^ Agency for Healthcare Research and Quality standard.

^b^ Number of cases excluded from overall IBD meta-analysis.

^c^ Number of cases included in overall IBD meta-analysis.

^d^ Pediatric-onset defined as 17 years or younger at diagnosis.

### Risk of Bias and Study Quality

Despite some variation in control of bias, all studies included were assessed to be of either fair or good quality (Table 2).^12,14,15,16,17^ All studies scored 3 or 4 (maximum score, 4) for selection, with the studies by El-Matary et al^12^ and Malham et al^15^ losing 1 point for not reporting exclusion of prevalent cancer cases at baseline and the study by Peneau et al^17^ losing 1 point for lack of description of the reference population, which was derived from the 1999 national population census with no further information on characteristics. All studies reported either matching at baseline or adjustment for confounders, with the exception of the study by Malham et al,^15^ which was awarded no points for comparability on this basis. All studies were awarded 2 points for assessment of outcome, but only the study by Olén et al^16^ was awarded 3 points for additionally reporting completeness in participant follow-up.

**Table 2.** Risk of Bias and Study Quality Assessment for Included Studies Using NOS

Source	Assessment of quality of a cohort study–NOS Domain^a^										Total NOS score (maximum, 9; AHRQ standard)
	Selection					Comparability and Outcome					
	Representativeness of exposed cohort	Selection of nonexposed cohort	Ascertainment of exposure	Demonstration that outcome of interest was not present at start of study	Total score (maximum, 4)	Comparability of cohorts on basis of design or analysis (maximum, 2)	Assessment of outcome	Was follow-up long enough for outcomes to occur? (≥6 mo)	Adequacy of follow-up of cohorts	Total score (maximum, 5)	
El-Matary et al,^12^ 2020	★ (a): All persons in University of Manitoba IBD Epidemiology Database who received diagnosis of IBD before age 18 y	★ (a): Manitoba universal health insurance	★ (a): Validated administrative case definition of IBD	(b): No statement of exclusion of prevalent cancer cases at point of follow-up	3	★ (a): Age, sex, and region of residence matched	★ (b): Linkage to Manitoba cancer registry	★ (a): More than 34-y study period	(d): No statement on adequacy of follow-up	3	6 (Good)
Kjaergaard et al,^14^ 2020	★ (a): All persons in Danish national patient registry who received diagnosis of IBD before age 18 y	★ (a): Reference population derived from Danish national patient registry	★ (a): *ICD-8* and *ICD-10* diagnosis; minimum 2 diagnoses from Danish national patient registry	★ (a): Individuals who received diagnosis of cancer before IBD diagnosis date were excluded	4	★★ (a): Reference population sex and age matched; confounders adjusted to include sex, age, and calendar year of diagnosis	★ (b): Linkage to Danish Cancer Registry	★ (a): More than 40-y study period	(d): No statement on adequacy of follow-up	4	8 (Good)
Malham et al,^15^ 2019	★ (a): All persons who received diagnosis of IBD from Special Reimbursement Registry (social insurance institution) before age 18 y	★ (a): Background national population in corresponding age groups	★ (a): *ICD-8*, *ICD-9*, and *ICD-10* diagnosis in Finnish national registry	(b): No statement of exclusion of prevalent cancer cases at point of follow-up	3	(c): No matching to reference population; no reporting of confounding	★ (b): Linkage to Finnish Cancer Registry; use of NORDCAN age-specific cancer rates for reference population	★ (a): Median 9.6-y follow-up time	(d): No statement on adequacy of follow-up	2	5 (Fair)
Olen et al,^16^ 2017	★ (a): All persons who received diagnosis of IBD in National Swedish Patient Register before age 18 y	★ (a): Derived from national Swedish Population Register	★ (a): *ICD-10* diagnosis in National Swedish Patient Register; minimum 2 diagnoses; diagnostic procedural codes (eg, disease-specific surgery)	★ (a): Individuals in pediatric IBD or general population who had cancer before start of follow-up were excluded	4	★★ (a): General population were matched by sex, age, year of birth, and county; confounders adjusted for were sex, age, birth year, and region of residence	★ (b): Linkage to National Swedish Cancer Register	★ (a): More than 50-y study period	★ (a): Estimated register completeness of >96%	5	9 (Good)
Peneau et al,^17^ 2013	★ (a): All persons in EPIMAD—retrospective population-based study of incidence cases of IBD in northern France since 1988 (<17 y)	(c): Reference population is not described	★ (b): Confirmation by 2 gastroenterologists; recorded as definite, probable, or possible	★ (a): No statement of exclusion of prevalent cancer cases at point of follow-up	3	★ (b): No matching to reference population; confounders adjusted to include age and sex	★ (b): Diagnosis from clinical record and confirmed using administrative health database	★ (a): Median 11.4-y follow-up time	(d): No statement on adequacy of follow-up	3	6 (Good)

Abbreviations: AHRQ, Agency for Healthcare Research and Quality; EPIMAD, Epidémiologie des maladies inflammatoires de l’Intestin; IBD, inflammatory bowel disease; *ICD-8*, *International Classification of Diseases, Eighth Revision*; *ICD-9*, *International Classification of Diseases, Ninth Revision*; *ICD-10*, *International Statistical Classification of Diseases and Related Health Problems, Tenth Revision*; NOS, Newcastle-Ottawa Scale; NORDCAN, Nordic Cancer Registry.

^a^ Each star totals 1 point on the NOS; (a), (b), (c), and (d) are NOS assessment form descriptors (available in eFigure 1 in the Supplement).

### Overall Cancer Rate

A total of 715 cancer cases were identified among 19 812 individuals with pediatric-onset IBD and 11 195 cancer cases were identified among 3 056 282 reference individuals. Total follow-up time of individuals with pediatric-onset IBD ranged from 8254 person-years in the study by Peneau et al^17^ to 148 682 person-years in the study by Olén et al.^16^ Incidence rates of cancer among patients with pediatric-onset IBD were reported in 4 studies^12,14,15,16^ and ranged from 1.0 to 3.3 per 1000 person-years.

**Figure 2.**
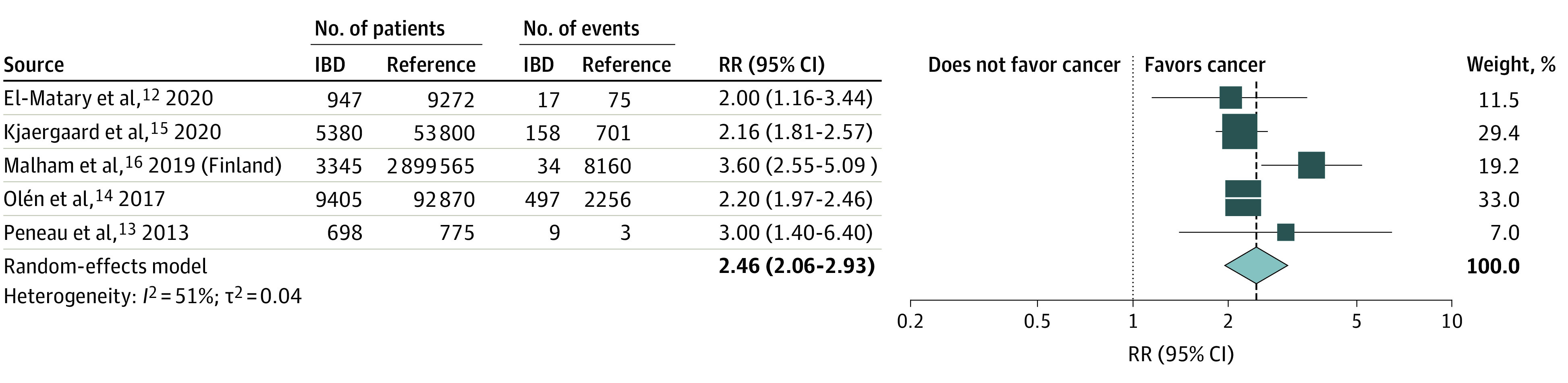
Forest Plot of Overall Meta-analysis of Relative Cancer Rate for Individuals With Pediatric-Onset Inflammatory Bowel Disease (IBD) Compared With Reference Populations RR indicates relative rate.

The overall pRR for cancer among individuals with pediatric-onset IBD compared with reference populations was 2.46 (95% CI, 2.06-2.93) (Figure 2). Tests for heterogeneity revealed *I*^2^ = 51% and τ^2^ = 0.04, indicating mild to moderate heterogeneity across included studies. The Swedish study by Olén et al^16^ included the largest number of patients, contributing a weight of 33.0% to the REM pRR.

Reported cancer rates among patients with pediatric-onset IBD and the reference populations are displayed in eFigure 1 in the Supplement. We observed the highest cancer occurrence in the Swedish study by Olén et al,^16^ with the lowest cancer rates for both the pediatric-onset IBD and reference populations seen in the Finnish data from Malham et al.^15^

### Cancer Rate Among Patients With CD or UC

**Figure 3.**
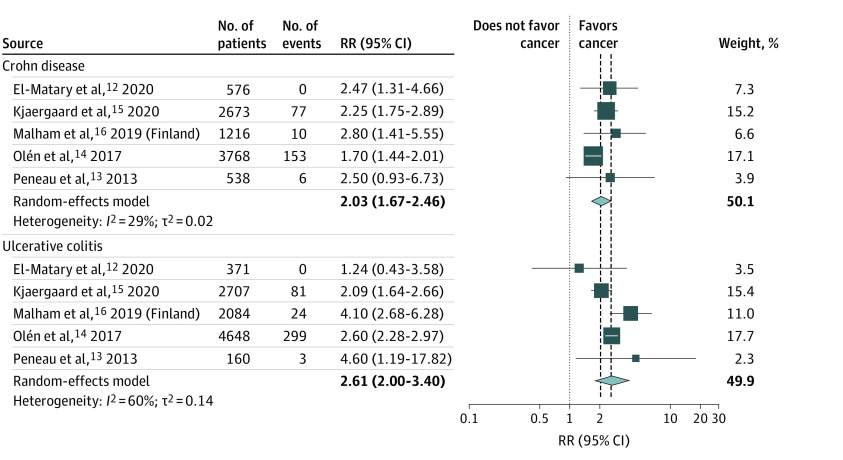
Forest Plot of Meta-analysis of Relative Cancer Rate for Individuals With Pediatric-Onset Inflammatory Bowel Disease (IBD) Compared With Reference Populations by IBD Subtype (Crohn Disease and Ulcerative Colitis) RR indicates relative rate.

All studies presented relative rate estimates for cancer by IBD subtype. The meta-analysis of pooled estimates by CD and UC shows a pRR of 2.03 (95% CI, 1.67-2.46) for CD and a pRR of 2.61 (95% CI, 2.00-3.40) for UC (Figure 3). Peneau et al^17^ reported a nonstatistically significantly increased rate for CD, as did El-Matary et al^12^ for UC, compared with their reference populations; both studies, however, contributed a small weight to the overall pooled summary statistic for the meta-analysis (7.0% and 11.5%, respectively).

### Cancer Rate According to Sex and Thiopurine Exposure

Only 2 studies report on the relative risk of cancer by sex.^14,16^ Meta-analysis of these studies revealed an increased relative rate of cancer among male patients with pediatric-onset IBD (pRR, 3.23; 95% CI, 2.35-4.45) and a nonsignificant increased relative rate for female patients with pediatric-onset IBD (pRR, 2.45; 95% CI, 0.93-6.46) compared with reference populations.

Data on the risk of cancer by treatment exposure were again presented only in 2 studies.^14,16^ Subgroup meta-analysis of these studies demonstrated an increased relative rate of cancer among patients treated with thiopurines (pRR, 2.09; 95% CI, 1.55-2.83), whereas the relative rate of cancer among patients not exposed to thiopurines was numerically but not statistically increased (pRR, 1.82; 95% CI, 0.63-5.22). However, estimates were similar, and 95% CIs were overlapping, indicating that the 2 estimates did not differ markedly from one another.

### Cancer Rate According to Site

Only 3 of the 5 studies consistently reported the relative cancer risk by cancer site among patients with pediatric-onset IBD.^14,15,16^ Meta-analyses of data from these 3 studies were undertaken for cancers specific to gastrointestinal sites, including colorectal cancers, small bowel cancers, liver cancers (cholangiocarcinomas and hepatocellular carcinomas), and cancers specific to extraintestinal sites, including lymphoid (leukemias and lymphomas), melanomas, and nonmelanoma skin cancers. The highest relative rates by cancer site were consistently observed for gastrointestinal cancers (eFigure 2 in the Supplement), with a 55-fold increased rate of liver cancer (pRR, 55.45; 95% CI, 19.59-156.99), followed by a 20-fold increased rate of colorectal cancer (pRR, 20.29; 95% CI, 15.90-25.90) and a 16-fold increased rate of small bowel cancer (pRR, 16.20; 95% CI, 3.52-74.66; eFigure 3 in the Supplement). The mean incidence rates of cancer by cancer site for these 3 studies indicate that, despite markedly increased relative rate estimates for gastrointestinal cancers among patients with pediatric-onset IBD compared with general pediatric populations, this risk corresponds to a mean incidence rate of 0.3 cases of liver cancer, 0.6 cases of colorectal cancer, and 0.1 cases of small bowel cancer per 1000 person-years in this population.

Estimates for the relative rate of extraintestinal cancers were lower, with the highest pRR seen for nonmelanoma skin cancer (pRR, 3.62; 95% CI, 1.97-6.66), followed by lymphoid cancer (pRR, 3.10; 95% CI, 1.88-5.10) and melanoma (pRR, 2.05; 95% CI, 1.27-3.29).

### Sensitivity Analysis and Publication Bias

A sensitivity meta-analysis excluding the study by Olén et al^16^ (the largest weighted study) and the study by Peneau et al^17^ (lowest contributor to overall REM weight) showed an increase in the pooled estimate (pRR, 2.52; 95% CI, 1.77-3.59; eFigure 4 in the Supplement). Inclusion of data from a non–peer-reviewed abstract by Deneau and Guthrey^19^ showed a further increased pRR of 2.95 (95% CI, 1.80-4.85; eFigure 5 in the Supplement). Both are small and nonsignificant increases to the overall pRR meta-analysis estimate. Overall, we found no indication of publication bias for the included studies (eFigure 6 in the Supplement).

## Discussion

In the present meta-analysis of available population-based cohort studies on the risk of cancer among patients with pediatric-onset IBD, including 19 812 patients with pediatric-onset IBD and 3 056 282 reference population individuals, we found a 2.4-fold increase in the relative rate of cancer among patients with pediatric-onset IBD compared with reference populations. Rates of cancer were similar among patients with CD and patients with UC and were highest among male patients. The increased rate of cancer among patients with pediatric-onset IBD was primarily associated with increased rates of gastrointestinal cancers. The association of thiopurine exposure with rates of cancer among patients with pediatric-onset IBD is unclear. The quality of the included studies was fair to good, and publication bias was not observed.

We restricted our meta-analysis to population-based studies to avoid selection bias and to minimize heterogeneity based on study design. The estimates reported in this study are consistent with findings from a systematic review by Aardoom et al,^6^ which included both selected and unselected studies and suggested an increased rate of cancer among patients with pediatric-onset IBD, with gastrointestinal cancers being the most frequently reported fatal cancer outcome.

On subgroup analysis, we found that the relative rate of cancer was similar among patients with CD and patients with UC, although numerically higher among those with UC. This finding may be associated with colorectal and hepatobiliary cancers, especially among patients with primary sclerosing cholangitis. Although the studies included in the meta-analysis did not consistently report this level of granularity, the particularly elevated pRR for liver cancers may be a reflection of increased risk of primary sclerosing cholangitis.

We were able to pool data from 2 studies^14,16^ on IBD medications and found that the relative rate of cancer among those exposed to thiopurines was twice as high as in the reference population, while the relative rate was 1.8-fold increased among those never exposed to thiopurines, although the latter estimate was not statistically significant. An increased rate of cancer after treatment with thiopurines has previously been suggested. Using data from the French national health insurance databases, Lemaitre et al^20^ reported an adjusted hazard ratio of 2.60 (95% CI, 1.96-3.44) for lymphoma among patients with IBD receiving thiopurine monotherapy compared with unexposed patients. Long et al^21^ reported that in a US health care claims database analysis, patients exposed to thiopurine therapy had higher odds of nonmelanoma skin cancer (adjusted odds ratio, 1.85; 95% CI, 1.66-2.05) but not melanoma (adjusted odds ratio, 1.10; 95% CI, 0.72-1.67). However, Kjaergaard et al^14^ and Olén et al^16^ did not find a significant association between thiopurine therapy and cancer, which could be owing to inadequate power to study this outcome, particularly given the limited use of thiopurines in pediatric populations.

We report a difference in overall relative cancer rate by sex, with the highest relative rate observed among male patients. A higher risk of lymphoma has previously been reported among young men receiving thiopurines,^22^ and male sex is considered a risk factor for cancer among children with IBD.^23^ Evidence increasingly suggests sex-based differences in response to IBD therapies^24^; these findings warrant further investigation.

Our findings indicate a particularly elevated relative rate of gastrointestinal cancers (including liver, small bowel, and colorectal cancers) among patients with pediatric-onset IBD compared with general pediatric populations. However, these estimates are derived from small numbers of cancer events in both IBD and non-IBD groups. The 55.5-fold increased relative rate of liver cancers identified here corresponds to an incidence of 0.3 cancer cases per 1000 person-years. The comparatively lower relative rate of extraintestinal cancers (including lymphoid and nonmelanoma and melanoma skin cancers) is in keeping with findings from Pedersen et al,^1^ who found a standardized incidence ratio of 1.1 (95% CI, 1.0-1.3) for extraintestinal cancer in a meta-analysis of 8 population-based cohort studies of patients with IBD.

Identifying variables that modulate cancer risk in pediatric patients would be valuable for targeting prevention and screening. For example, ongoing inflammation is an important risk factor for cancer, specifically gastrointestinal cancer, and early and adequate control of inflammation is critical to preventing long-term complications.^24^ Guidance on screening for colorectal cancer among children is similar to that for adults; a colonoscopy is recommended 6 to 8 years after diagnosis for patients with colitis extending beyond the rectum, and an annual colonoscopy is recommended from the time of diagnosis for patients with primary sclerosing cholangitis.^25^ Annual screening for skin cancer is currently recommended for all patients with IBD.^26^

### Strengths and Limitations

This study has some strengths; the primary strength is the careful inclusion of population-based studies only (ie, high-quality unselected studies representing the entire population of patients with pediatric-onset IBD). Determining a pooled estimate of relative cancer rate based on high-quality studies is novel and has important clinical implications. This decreases the likelihood of selection bias and ensures generalizability of results. Accordingly, the heterogeneity scores in our analyses are moderate to low. Although all of the included studies were assessed as fair to good using Newcastle-Ottawa Scale scoring, this quality assessment tool has limitations for assessing the internal validity of the studies, particularly when being applied to a selection of high-quality studies with good control for bias, as is the case with the studies included in this meta-analysis.^27^

This study also has some limitations, including a lack of data on disease severity and limited data on exposure to thiopurines and biologic therapies. Inconsistent data on cohort characteristics (eg, age at IBD diagnosis or cancer diagnosis) and a lack of granular data on subtypes of cancer are also limitations.All of the data identified for analysis are based in high-income, Western populations, so the findings might not be generalizable beyond similar populations.

## Conclusions

In this meta-analysis, we found a 2.4-fold increase in the relative rate of cancer among patients with pediatric-onset IBD compared with the general pediatric population, primarily associated with an increased rate of gastrointestinal cancers. Although the incidence of pediatric-onset IBD is increasing, the overall incidence rate of cancer in this population is low (ie, <3.3 cases per 1000 person-years).

## Notes

Supplement. **eTable 1.** Embase Database Search Using Ovid **eTable 2.** MEDLINE (Pubmed With MeSH Headings) Search Terms **eFigure 1.** Newcastle-Ottawa Scale Tool Description for Assessment of Risk of Bias and Study Quality **eFigure 2.** Forest Plots of Meta-Analyses for Relative Cancer Risk for Individuals With Pediatric-Onset IBD Compared With Reference Populations by Gastrointestinal Cancer **eFigure 3.** Forest Plots of Meta-Analyses for Relative Cancer Risk for Individuals With Pediatric-Onset IBD Compared With Reference Populations by Extra-Intestinal Cancer **eFigure 4.** REM Meta-Analysis of Cancer Rates in Pediatric-Onset IBD Compared With Reference Populations Excluding Olen et al. and Penau et al. **eFigure 5.** REM Meta-Analysis of Cancer Rates in Paediatric-Onset IBD Compared With Reference Populations Including Deneau & Guthery, 2017 **eFigure 6.** Funnel Plot for Assessment for Publication Bias of Included Studies
