# Supplementary material for: Development of Cancer Among Patients With Pediatric-Onset Inflammatory Bowel Disease: A Meta-analysis of Population-Based Studies
Source: JAMA Netw Open. 2022 Mar 1;5(3):e220595. doi: 10.1001/jamanetworkopen.2022.0595 (PMC8889462; doi:10.1001/jamanetworkopen.2022.0595)
Supplement: Supplement. — eTable 1. Embase Database Search Using Ovid eTable 2. MEDLINE (Pubmed With MeSH Headings) Search Terms eFigure 1. Newcastle-Ottawa Scale Tool Description for Assessment of Risk of Bias and Study Quality eFigure 2. Forest Plots of Meta-Analyses for Relative Cancer Risk for Individuals With Pediatric-Onset IBD Compared With Reference Populations by Gastrointestinal Cancer eFigure 3. Forest Plots of Meta-Analyses for Relative Cancer Risk for Individuals With Pediatric-Onset IBD Compared With Reference Populations by Extra-Intestinal Cancer eFigure 4. REM Meta-Analysis of Cancer Rates in Pediatric-Onset IBD Compared With Reference Populations Excluding Olen et al. and Penau et al. eFigure 5. REM Meta-Analysis of Cancer Rates in Paediatric-Onset IBD Compared With Reference Populations Including Deneau & Guthery, 2017 eFigure 6. Funnel Plot for Assessment for Publication Bias of Included Studies [file jamanetwopen-e220595-s001.pdf]

Supplementary Online Content

Elmahdi R, Lemser CE, Thomsen SB, Allin KH, Agrawal M, Jess T. Development of cancer among patients with pediatric-onset inflammatory bowel disease: a meta-analysis of population-based studies. *JAMA Netw Open.* 2022;5(3):e220595. doi:10.1001/jamanetworkopen.2022.0595

eTable 1. Embase Database Search Using Ovid

eTable 2. MEDLINE (Pubmed With MeSH Headings) Search Terms

eFigure 1. Newcastle-Ottawa Scale Tool Description for Assessment of Risk of Bias and Study Quality

eFigure 2. Forest Plots of Meta-Analyses for Relative Cancer Risk for Individuals With Pediatric-Onset IBD Compared With Reference Populations by Gastrointestinal Cancer

eFigure 3. Forest Plots of Meta-Analyses for Relative Cancer Risk for Individuals With Pediatric-Onset IBD Compared With Reference Populations by Extra-Intestinal Cancer

eFigure 4. REM Meta-Analysis of Cancer Rates in Pediatric-Onset IBD Compared With Reference Populations Excluding Olen et al. and Penau et al.

eFigure 5. REM Meta-Analysis of Cancer Rates in Paediatric-Onset IBD Compared With Reference Populations Including Deneau & Guthery, 2017

eFigure 6. Funnel Plot for Assessment for Publication Bias of Included Studies

This supplementary material has been provided by the authors to give readers additional information about their work.

© 2022 Elmahdi R et al. *JAMA Network Open.*

eTable 1. Embase Database Search Using Ovid

Search Terms (Embase Ovid)				
	**1**	**2**	**3**	**4**
Search Terms	Malignant neoplasm	Inflammatory Bowel Diseases	Pediatrics<br>Juvenile<br>Adolescent<br>Child	Epidemiology<br>Cohort analysis
Free text	Cancer*<br>Neoplasm*<br>Neoplasia<br>Adenocarcinoma<br>Carcinoma<br>Malignancy<br>Tumour* / Tumor*	Inflammatory Bowel Disease*<br>IBD*<br>Crohn<br>Crohn's Disease<br>Morbus Crohn<br>Colitis Ulcerosa<br>Ulcerative colitis	Pediatric / Paediatric<br>Early onset / Early-onset<br>Children<br>Child<br>Adolescent<br>Juvenile<br>Infant	Cohort analysis<br>Cohort study<br>Population based

© 2022 Elmahdi R et al. *JAMA Network Open.*

eTable 2. MEDLINE (Pubmed With MeSH Headings) Search Terms

Subject headings (MEDLINE PubMed)				
	**1**	**2**	**3**	**4**
Subject headings	Malignant neoplasm	Inflammatory Bowel Diseases	Pediatrics<br>Juvenile<br>Adolescent<br>Child	Epidemiology<br>Cohort analysis
Free text	(“Neoplasms”[MeSH] OR Cancer* OR Neoplasia OR Adenocarcinoma OR Carcinoma OR Malignancy OR Tumour* OR Tumor*)	(“Inflammatory Bowel Diseases”[MeSH] OR “Inflammatory Bowel Disease*” OR IBD* OR Crohn OR “Crohn’s Disease” OR “Morbus Crohn” OR “Colitis Ulcerosa” OR “Ulcerative colitis”)	(“Infant”[MeSH] OR “Child, Preschool”[MeSH] OR “Child”[MeSH] OR “Adolescent”[MeSH] OR Pediatric OR Paediatric OR Early onset OR Early-onset OR Children OR Child OR Adolescent OR Juvenile OR Infant)	(“Epidemiology”[MeSH] OR “Cohort analysis”[MeSH] OR Cohort analysis OR Cohort study OR Population based)

© 2022 Elmahdi R et al. *JAMA Network Open.*

RISK OF BIAS AND QUALITY ASSESSMENT:

eFigure 1. Newcastle-Ottawa Scale Tool Description for Assessment of Risk of Bias and Study Quality

Newcastle-Ottawa Quality Assessment Form for Cohort Studies

Note: A study can be given a maximum of one star for each numbered item within the Selection and Outcome categories. A maximum of two stars can be given for Comparability.

Selection

1) Representativeness of the exposed cohort
   a) Truly representative ***(one star)***
   b) Somewhat representative ***(one star)***
   c) Selected group
   d) No description of the derivation of the cohort
2) Selection of the non-exposed cohort
   a) Drawn from the same community as the exposed cohort ***(one star)***
   b) Drawn from a different source
   c) No description of the derivation of the non exposed cohort
3) Ascertainment of exposure
   a) Secure record (e.g., surgical record) ***(one star)***
   b) Structured interview ***(one star)***
   c) Written self report
   d) No description
   e) Other
4) Demonstration that outcome of interest was not present at start of study
   a) Yes ***(one star)***
   b) No

Comparability

1) Comparability of cohorts on the basis of the design or analysis controlled for confounders
   a) The study controls for age, sex and marital status ***(one star)***
   b) Study controls for other factors (list) ______________________________ ***(one star)***
   c) Cohorts are not comparable on the basis of the design or analysis controlled for confounders

Outcome

1) Assessment of outcome
   a) Independent blind assessment ***(one star)***
   b) Record linkage ***(one star)***
   c) Self report
   d) No description
   e) Other
2) Was follow-up long enough for outcomes to occur
   a) Yes ***(one star)***
   b) No

Indicate the median duration of follow-up and a brief rationale for the assessment above:____________________

3) Adequacy of follow-up of cohorts
   a) Complete follow up- all subject accounted for ***(one star)***
   b) Subjects lost to follow up unlikely to introduce bias- number lost less than or equal to 20% or description of those lost suggested no different from those followed. ***(one star)***
   c) Follow up rate less than 80% and no description of those lost
   d) No statement

© 2022 Elmahdi R et al. *JAMA Network Open.*

Thresholds for converting the Newcastle-Ottawa scales to AHRQ standards (good, fair, and poor):

Good quality: 3 or 4 stars in selection domain AND 1 or 2 stars in comparability domain AND 2 or 3 stars in outcome/exposure domain

Fair quality: 2 stars in selection domain AND 1 or 2 stars in comparability domain AND 2 or 3 stars in outcome/exposure domain

Poor quality: 0 or 1 star in selection domain OR 0 stars in comparability domain OR 0 or 1 stars in outcome/exposure domain

© 2022 Elmahdi R et al. *JAMA Network Open.*

META-ANALYSES

eFigure 2. Forest Plots of Meta-Analyses for Relative Cancer Risk for Individuals With Pediatric-Onset IBD Compared With Reference Populations* by Gastrointestinal Cancer

A. Colorectal cancer, B. Small bowel cancer, and C. Liver cancers

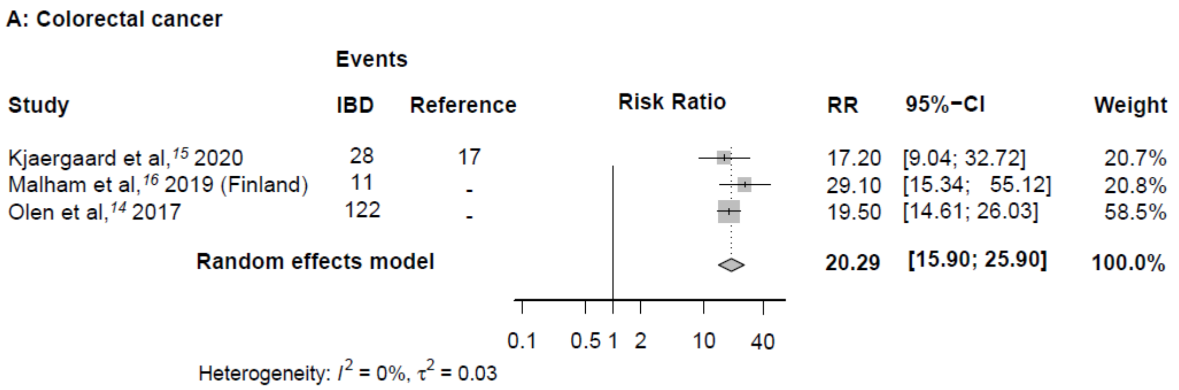

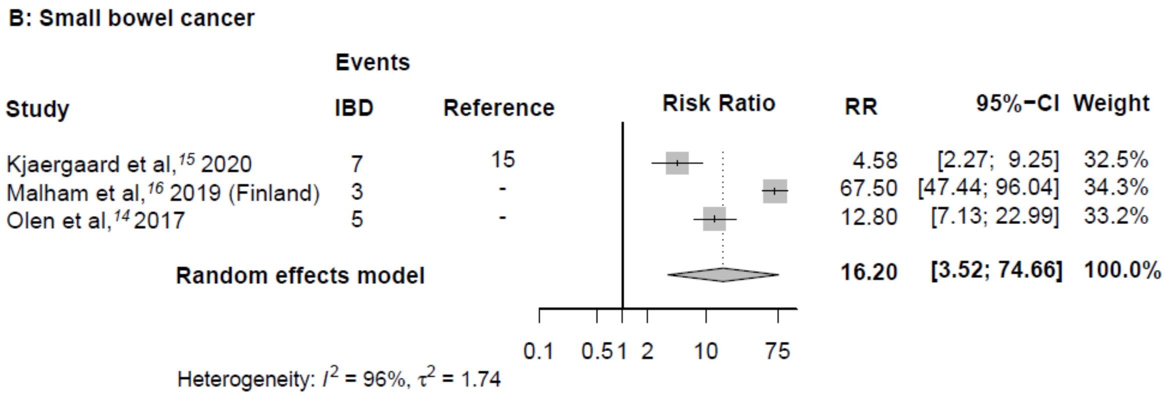

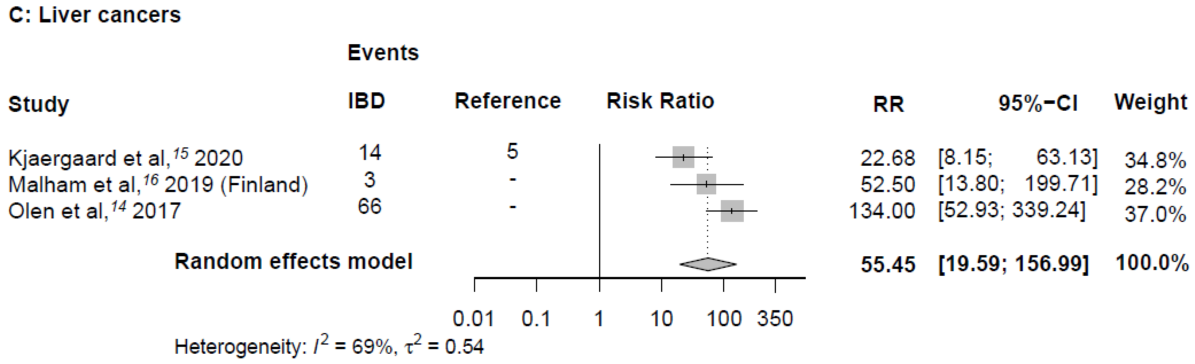

*Including data from Kjaergaard et al., Malham et al. and Olen et al.

© 2022 Elmahdi R et al. *JAMA Network Open.*

eFigure 3. Forest Plots of Meta-Analyses for Relative Cancer Risk for Individuals With Pediatric-Onset IBD Compared With Reference Populations* by Extra-Intestinal Cancer

A. Lymphoid cancers, B. Non-melanoma skin cancer and C. Melanoma skin cancer

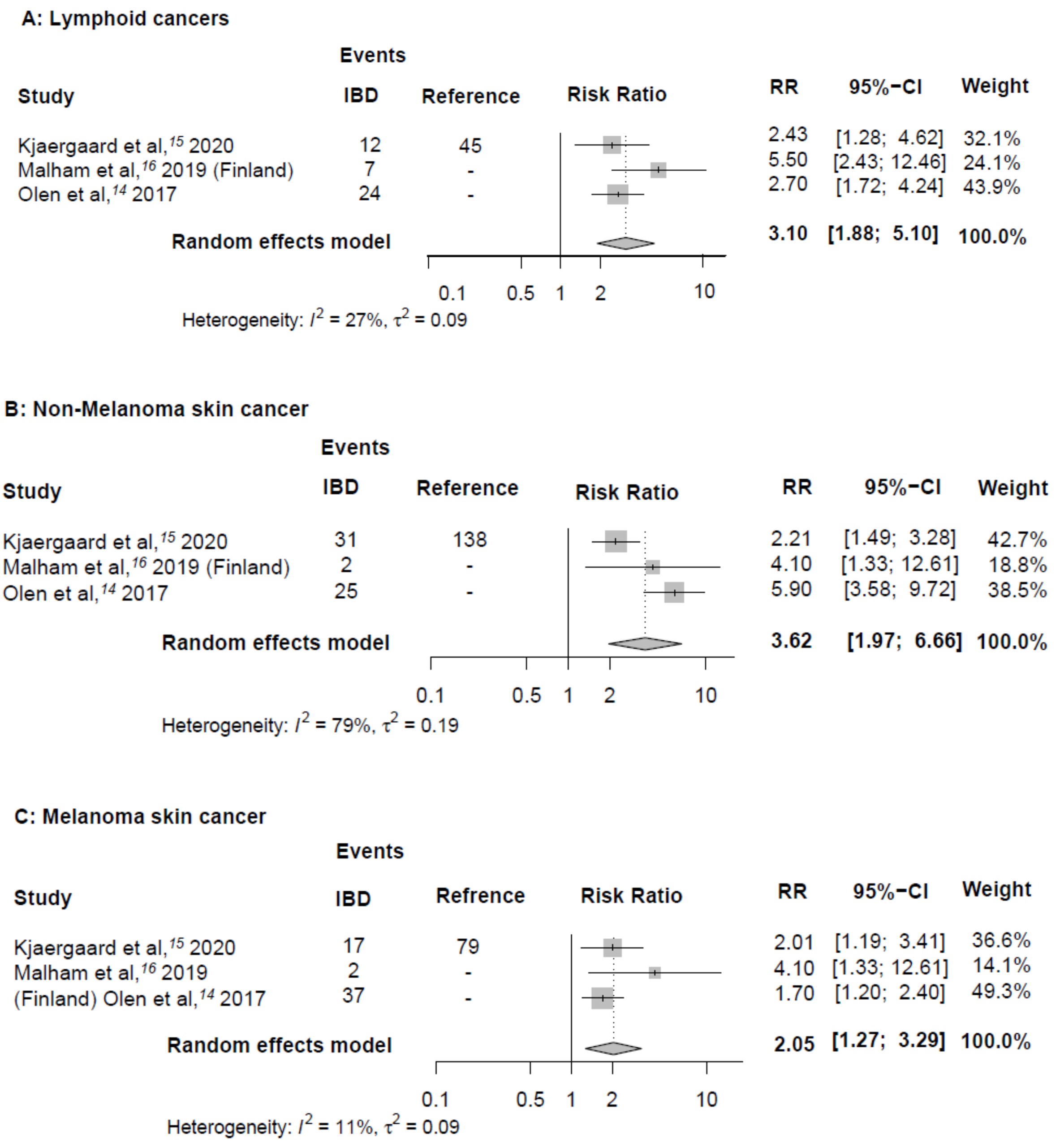

*Including data from Kjaergaard et al., Malham et al. and Olen et al.

© 2022 Elmahdi R et al. *JAMA Network Open.*

eFigure 4. REM Meta-Analysis of Cancer Rates in Pediatric-Onset IBD Compared With Reference Populations Excluding Olen et al. and Penau et al.

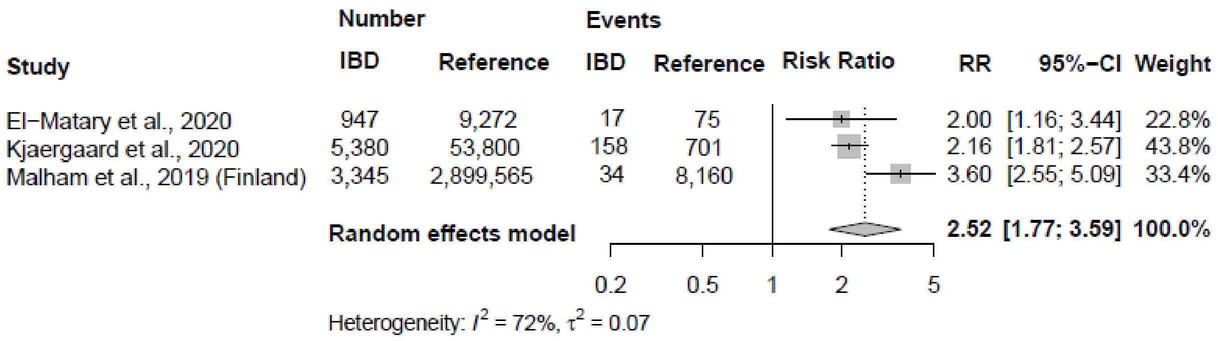

© 2022 Elmahdi R et al. *JAMA Network Open.*

eFigure 5. REM Meta-Analysis of Cancer Rates in Paediatric-Onset IBD Compared With Reference Populations Including Deneau & Guthery, 2017

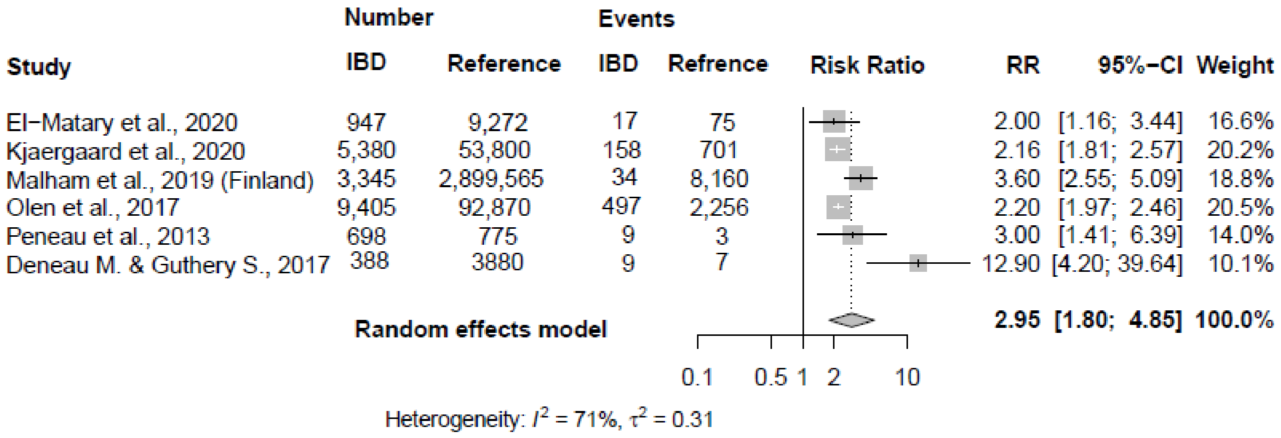

© 2022 Elmahdi R et al. *JAMA Network Open.*

eFigure 6. Funnel Plot for Assessment for Publication Bias of Included Studies

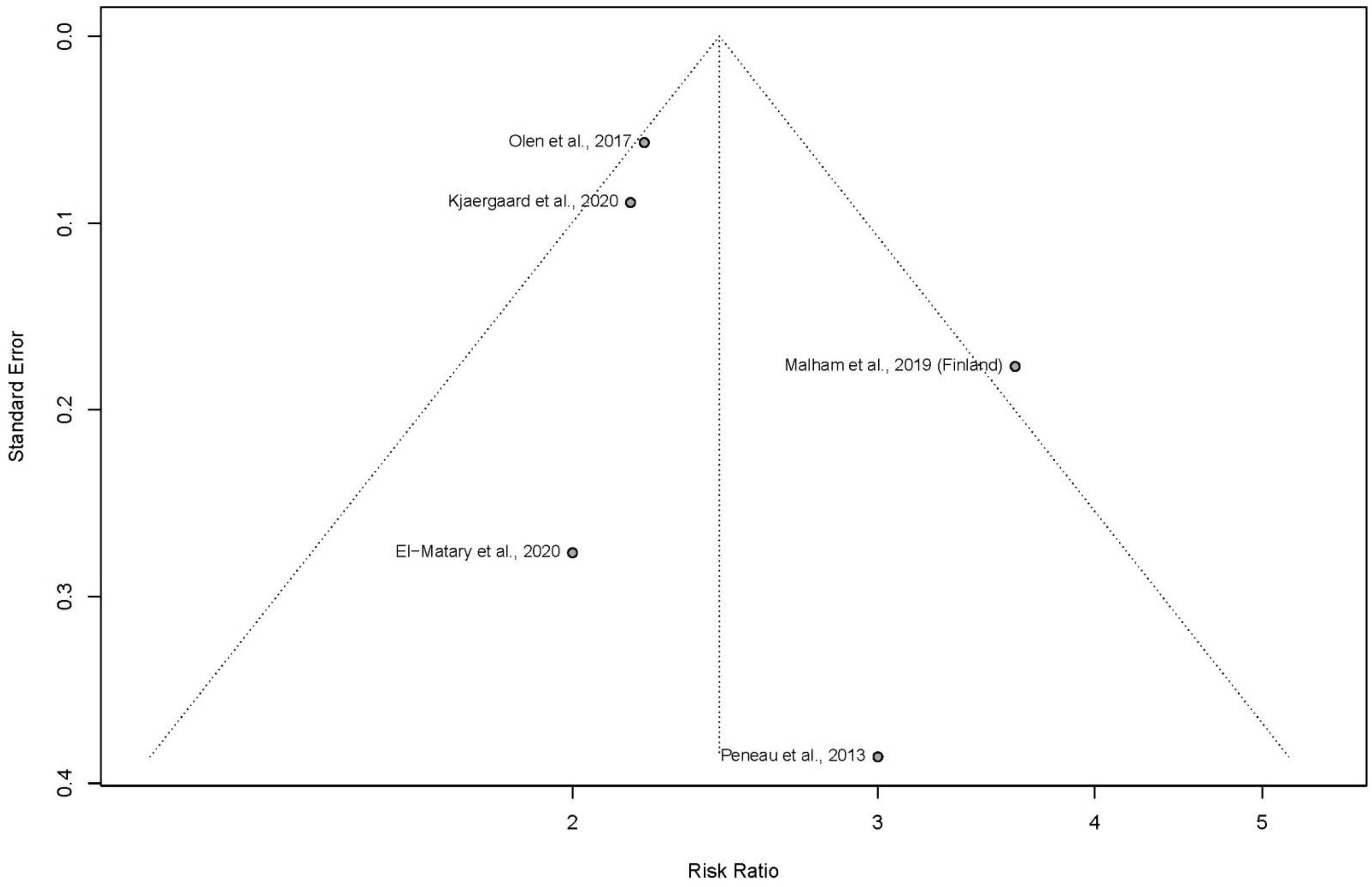

© 2022 Elmahdi R et al. *JAMA Network Open.*
